# Neurexin and Neuroligins Maintain the Balance of Ghost and Satellite Boutons at the *Drosophila* Neuromuscular Junction

**DOI:** 10.3389/fnana.2020.00019

**Published:** 2020-06-09

**Authors:** Gan Guangming, Geng Junhua, Zhang Chenchen, Mou Yang, Xie Wei

**Affiliations:** ^1^School of Medicine, Southeast University, Nanjing, China; ^2^The Key Laboratory of Developmental Genes and Human Disease, Institute of Life Sciences, Southeast University, Nanjing, China; ^3^Institute of Life Sciences, The Collaborative Innovation Center for Brain Science, Southeast University, Nanjing, China

**Keywords:** neurexin, neuroligins, *Drosophila*, neuromuscular junction, ghost bouton, satellite boutons

## Abstract

Neurexins and neuroligins are common synaptic adhesion molecules that are associated with autism and interact with each other in the synaptic cleft. The *Drosophila* neuromuscular junction (NMJ) bouton is a well-known model system in neuroscience, and ghost and satellite boutons, respectively, indicate the poor development and overgrowth of the NMJ boutons. However, the *Drosophila* neurexin (DNrx) and *Drosophila* neuroligins (DNlgs) are mainly observed in type Ib boutons, indicating the ultrastructural and developmental phenotypes of the *Drosophila* NMJ. Here, we identified the ultrastructural and developmental features of ghost and satellite boutons by utilizing *dneurexin* (*dnrx*) and *dneuroligins* (*dnlgs*) fly mutants and other associated fly strains. Ghost boutons contain synaptic vesicles with multiple diameters but very rarely contain T-bar structures and swollen or thin subsynaptic reticulum (SSR) membranes. The muscle cell membrane is invaginated at different sites, stretches to the ghost bouton from different directions, forms several layers that enwrap the ghost bouton, and then branches into the complex SSR. Satellite boutons share a common SSR membrane and present either a typical profile in which a main bouton is encircled by small boutons or two atypical profiles in which the small boutons are grouped together or distributed in beads without a main bouton. Electron and confocal microscopy data showed that *dnrx*, *dnlg1*, *dnlg2*, *dnlg3*, and *dnlg4* mutations led to ghost boutons; the overexpression of *dnrx*, *dnlg1*, *dnlg2*, *dnlg3*, and *dnlg4* led to satellite boutons; and the *dnlg2;dnlg3* double mutation also led to satellite boutons. These results suggested that DNrx and DNlgs jointly maintain the development and function of NMJ boutons by regulating the balance of ghost and satellite boutons in *Drosophila*.

## Introduction

Neurexins and neuroligins are pre- and postsynaptic adhesion molecules, respectively, that are associated with autism. Caki interacts with neurexin before synapse formation and is involved in synaptic development (Sun et al., 2009), and neurexins and neuroligins interact with each other in the synaptic cleft. Moreover, *dnrx* (Li et al., 2007; Rui et al., 2017), *dnlg1* (Banovic et al., 2010), *dnlg2* (Sun et al., 2009, 2011), *dnlg3* (Xing et al., 2014), and *dnlg4* (Li et al., 2013; Zhang et al., 2017) are reportedly involved in synapse formation and synaptic transmission in *Drosophila* neuromuscular junction (NMJ) type I boutons. Furthermore, *dnrx* (Larkin et al., 2015; Tong et al., 2016) and *dnlg4* (Li et al., 2013) were shown to disrupt *Drosophila* sleep patterns. Research based on the *Drosophila* larval NMJ model showed that the number of synaptic boutons was reduced in *dnrx* (Li et al., 2007; Rui et al., 2017), *dnlg1* (Banovic et al., 2010), *dnlg2* (Sun et al., 2009, 2011), and *dnlg4* (Li et al., 2013; Zhang et al., 2017) mutants, and this phenotype could be rescued in all mutants by a gene overexpression system. The number of synaptic boutons increased and could also be rescued in the *dnlg3* mutant (Xing et al., 2014). Furthermore, the number of synaptic boutons increased in overexpression strains of *dnrx* (Li et al., 2007; Rui et al., 2017) and *dnlg2* (Sun et al., 2009, 2011) and decreased in the *dnlg3* (Xing et al., 2014) overexpression strain. These results showed that *dnrx*, *dnlg1*, *dnlg2*, *dnlg3*, and *dnlg4* together maintain the number of type I boutons.

The *Drosophila* larval NMJ is a well-known model system for studying synaptic development, signal transmission, and neurological disease and is classified by three distinct types of synaptic boutons (types I, II, and III) according to size, subsynaptic reticulum (SSR) characteristics, neurotransmitter identity, and synaptic vesicle composition in wild-type organisms (Atwood et al., 1993; Jia et al., 1993; Monastirioti et al., 1995). Type I boutons, the main type in the sixth/seventh muscles, have been studied in research on gene functions and neurological disorders, such as spinocerebellar ataxia (Tsuda et al., 2005), amyotrophic lateral sclerosis (Chai et al., 2008; Tsuda et al., 2008), spinal muscular atrophy (Sen et al., 2011), and Alzheimer’s disease (Zempel and Mandelkow, 2015). In type I boutons, abnormal morphological phenotypes are mainly characterized by two manifestations on confocal and transmission electron microscopy (TEM). The first phenotype constitutes abnormal ultrastructural characteristics, such as abnormalities in the SSR (Banerjee et al., 2017), synapse and T-bar structures (Xing et al., 2014), and synaptic vesicles (Xing et al., 2014). The other phenotype is the appearance of two types of abnormal boutons, namely, ghost boutons and satellite boutons, on confocal microscopy.

On confocal microscopy, ghost boutons contain synaptic vesicles but no active zones. These structures express a neuronal membrane marker recognized by the anti-HRP antibody (Sutcliffe et al., 2013) but lack the postsynaptic Discs-large (Dlg) protein and GluRs (Ataman et al., 2006; Menon et al., 2013; Sutcliffe et al., 2013; Piccioli and Littleton, 2014). Ghost boutons are associated with poor synaptic development (Sutcliffe et al., 2013). Satellite boutons are constituted by several small boutons that bud from a parent bouton present in a branch of the terminal arbor (Lee and Wu, 2010), however, in wild-type larval NMJs, a branching parent bouton normally has at most two new branches (Miller et al., 2012). Satellite boutons have also been shown to bud from an axonal segment that connects two adjacent boutons (Torroja et al., 1999). Satellite boutons contain the presynaptic proteins Synapsin and Brp, and the postsynaptic proteins Dlg and GluRs are more common in mutants that display NMJ bouton overgrowth than in the wild type (Dickman et al., 2006). Satellite boutons are correlated with genes associated with human mental illnesses (Endris et al., 2002; Fuentes-Medel et al., 2009; Li et al., 2016).

Reports of ghost and satellite boutons are principally based on laser scanning confocal microscopy, which has low resolution, and only limited TEM ultrastructural data are available. Therefore, the ultrastructural phenotypes of the common synaptic adhesion molecules *Drosophila* neurexin (DNrx) and *Drosophila* neuroligins (DNlgs) are mainly determined from type Ib boutons. Because NMJ boutons are fairly diffuse in muscles and because the muscles in *Drosophila* larvae are often curled, researchers must prepare many slices to obtain sufficient NMJ bouton samples. In this study, we prepared a wide range of samples and analyzed the morphological properties and development of ghost and satellite boutons in *Drosophila* larvae with *caki*, *dnrx*, and *dnlgs* mutants, the wild-type strain and a *dbrat* mutant with typical satellite boutons on confocal microscopy (Shi et al., 2013). The results of this study suggested that DNrx and DNlgs jointly maintain the balance of ghost and satellite boutons in the *Drosophila* NMJ.

## Materials and Methods

### *Drosophila* Stocks

The *w*^1118^ strain was used as the wild-type control in this study. All stocks were cultured in standard medium at 25°C. Fly stocks Df(3R) X-313 (hereafter called *caki*^313^) and Df(3R)X-307 (hereafter called *caki*^307^) were obtained from the Bloomington Stock Center at Indiana University (Bloomington, IN, United States), and both strains were shown to carry a recessive lethal partial deletion of the *caki* gene (Sun et al., 2009). The *dbrat*^11^ and *dbrat*^192^ strains were obtained from Dr. Zhang and were previously shown to carry a recessive lethal partial deletion of the *dbrat* gene (Shi et al., 2013). The following fly mutants were used: *dnrx*^273^ (Li et al., 2007), *dnlg1*^*ex*1.9^ and *dnlg1*^*ex*2.3^ (Banovic et al., 2010), *dnlg2*^*KO*70^ and *dnlg3*^*KO*127^ (Xing et al., 2018), and *dnlg4*^KO10^ (Zhang et al., 2017). The following *w*^1118^ overexpression strains were used: *24B-Gal4;UAS-dnlg1*, *24B-Gal4;UAS-dnlg2*, *MHC-Gal4;UAS-dnlg3* (Xing et al., 2014), and *Ok6-Gal4;UAS-dnlg4* (Zhang et al., 2017), as well as *dnlg2*^*KO*70^;*dnlg3*^*KO*127^, *Ok6-Gal4;UAS-dnrx*, and *MHC-Gal4;UAS-dnlg1*, which were generated in our laboratory. The rescue strains were *24B-Gal4;UAS-dnlg2* (Sun et al., 2011) and *Elav-Gal4;UAS-dnlg3* (Xing et al., 2014).

### TEM of Larval NMJ Boutons

Dissection and fixation were based on standard procedures (Xing et al., 2018). In brief, the wandering late third-instar larvae were dissected with standard techniques in Jan solution (128 mM NaCl, 2 mM KCl, 4 mM MgCl_2_, 35 mM sucrose, 5 mM HEPES, pH 7.4) and fixed at 4°C overnight in a mixture of 2% glutaraldehyde and 2% formaldehyde in 0.1 M sodium cacodylate buffer (pH 7.4), followed by several rinses with cacodylate buffer. The samples were then postfixed for 2 h with 1% OsO_4_ in cacodylate buffer and rinsed twice with distilled water. The preparations were stained for 2 h with 2% saturated uranyl acetate in distilled water and rinsed twice with distilled water. The specimens were dehydrated in an ethanol series, passed through propylene oxide two times, and embedded into a sheet in Epon812 (SPI Science). A total of 80 microns was serially sectioned using a diamond knife on a Leica UC7 ultrathin microtome at the sixth/seventh muscles of the A_3_ or A_2_ segment in one animal; each slice was 90 nm thick, 30–40 slices were gathered into a group and attached to a grid, and approximately 30 grids were used. The grids were poststained with 2% saturated uranyl acetate in 50% ethanol and 1% lead citrate (pH 12) and examined under a transmission electron microscope (Hitachi H-7650). More than 20 wild-type animals were analyzed, and 3 animals were analyzed for the other strains.

### The Procedure for Pre-embedding Immunogold Electron Microscopy

Pre-embedding immunogold electron microscopy was performed based on standard procedures (Gan and Zhang, 2018). In brief, wandering late third-instar larvae were dissected and fixed in a mixed agent (4% formaldehyde, 0.5% glutaraldehyde, and 10% saturated picric acid in 0.1 M sodium cacodylate buffer, pH 7.4) for 4 h. Then, the samples were washed with 0.1 M sodium cacodylate buffer diluted with 1% saponin for 1 h, preincubated in 0.1% BSA with 0.1% saponin for 1 h, and incubated with a mouse anti-Dlg primary antibody (DSHB) for 24 h at 4°C. After four rinses with 0.1% Tween-20, the samples were preincubated with 0.1% gelatin, 0.5% BSA and 0.1% saponin in 0.1 M PBS for 1 h, incubated with a 1.4 nm ultrasmall gold-conjugated secondary antibody (goat anti-mouse IgG secondary antibody, Nanoprobes, #2001, 1:50) for 1 h at 4°C, and rinsed four times with 0.1% Tween-20 in 0.1 M PBS. The samples were then postfixed in 2.0% glutaraldehyde in PBS for 30 min and rinsed several times with distilled water. Silver enhancement (HQS kit; Nanoprobes, #2012) was performed in the dark at 4°C for 25 min, followed by rinsing with distilled water. After rinsing with PBS for 10 min, the samples were either osmicated in 0.1 M sodium cacodylate for 1 h or not. All samples were washed three times with distilled water and then stained with 2% aqueous uranyl acetate for 2 h at 4°C. Subsequent epoxy resin embedding, trimming, and thin sectioning were performed as described above for the NMJ boutons at the sixth/seventh muscles in the A_3_ or A_2_ segment.

### Immunochemistry

Immunostaining of the larval samples was performed as described previously (Xing et al., 2014). Briefly, the larval samples were fixed for 40 min in paraformaldehyde, washed four times with PBS and 0.3% PBST (0.3% Triton X-100 in PBS), blocked in 1% bovine serum albumin for 1 h, incubated with anti-Hrp (Jackson ImmunoResearch, West Grove, PA, United States) or anti-Dlg (4F3; 1:50; DSHB) at 4°C for 2 h, and incubated with fluorophore-conjugated secondary antibodies (Invitrogen, 1:500) for 1 h at room temperature. The samples were washed extensively and mounted in VectaShield mounting medium (Vector Laboratories). The images were collected using an Olympus FV3000 confocal microscope.

### Statistical Analysis

The ghost and satellite boutons were counted at the sixth/seventh muscles of the A2–A3 segments from *Drosophila* larvae. As to each strain, at least eight segments were counted for confocal microscopy data, and at least three segments were counted for TEM data. The data were analyzed with GraphPad Prism 5 using unpaired, two-tailed *t*-tests.

## Results

### Identification of Ghost Boutons in Wild-Type Flies by TEM

**FIGURE 1:**
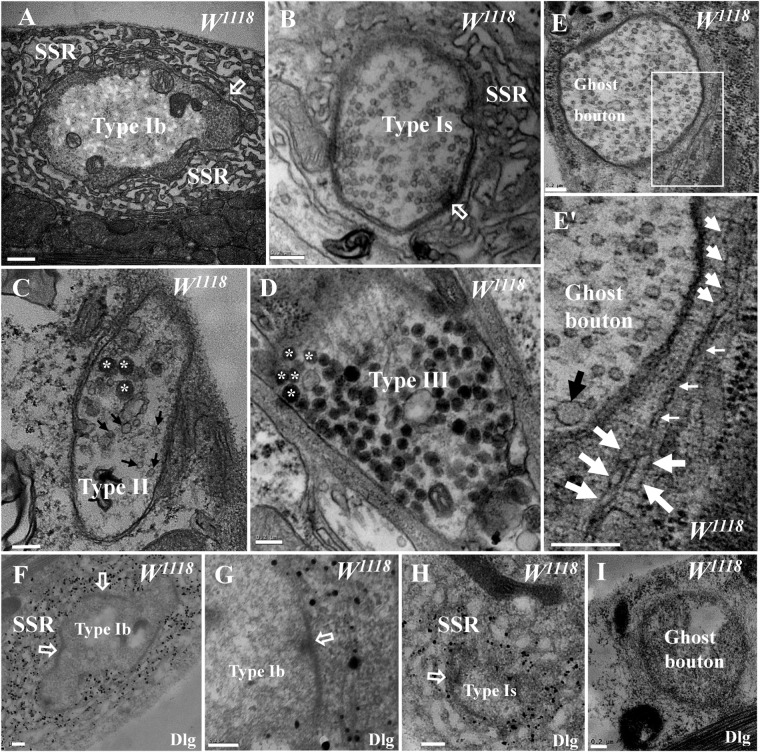
General characteristics of type I, type II, type III, and ghost boutons in wild-type samples. A type Ib bouton with a thick subsynaptic reticulum (SSR) and T-bar **(A)** and a type Is bouton with a less developed SSR and T-bar **(B)**. A type II bouton contains dense core vesicles and clear vesicles without an SSR **(C)**, and a type III bouton contains dense core vesicles without an SSR **(D)**. Ghost boutons have no T-bar structures or SSR membranes **(E)** with large synaptic vesicles **(E′)** and rare swollen SSR membranes **(E′)**. Dlg signals (black dots) were dense in the SSR membranes of type Ib boutons **(F,G)** and sparse in type Ib boutons **(H)** but not in ghost boutons **(I)** or in the first muscle cell membrane of type Ib boutons **(G)**. Hollow arrows show T-bars and synapses, large black arrows show large clear vesicles, small black arrows show small clear vesicles, and asterisks indicate dense core vesicles. Thick white arrows show swollen SSR membranes, and thin white arrows show thin SSR membranes. **(E′)** is an enlargement of the white box in **(E)**. Scale bars: **(A)**, 500 nm; **(B–I)**, 200 nm.

*Drosophila* larval NMJs were classified as type I, type II, and type III boutons according to their size, SSR characteristics, and synaptic vesicle composition under TEM (Atwood et al., 1993; Jia et al., 1993). The type Ib bouton contained a thick SSR with a large size (Figure 1A), and the type Is bouton had a less developed SSR with a small size (Figure 1B) in the wild-type flies. Both type II and type III boutons lacked the distinctive SSR, type II boutons contained both dense core vesicles and small clear vesicles (Figure 1C), and type III boutons mainly contained dense core vesicles (Figure 1D).

Ghost boutons are extremely rarely observed under confocal microscopy, and the current literature has not described these structures in wild-type *Drosophila* under TEM. We found ghost boutons in wild-type *Drosophila* in a wide range of serial sections from more than 20 animals. The ghost boutons had no synapses, T-bar structures, or SSR membranes (Figure 1E) but had synaptic vesicles with various diameters (Figure 1E′). There were two layers of paired membranes in the ghost boutons: the inner membrane was from the axon terminal, and the other membrane was from the muscle cell membrane. The ghost boutons were extremely rarely observed with TEM, and we observed only one ghost bouton between the sixth and seventh muscles in more than five animals. Occasionally, two SSR membrane layers were observed to stretch along a ghost bouton, and the SSR membranes did not branch (Figure 1E′). A considerable fraction of the SSR membranes were swollen (Figure 1E′).

Dlg is widely used as a conventional molecular marker of postsynaptic SSR membranes in *Drosophila* NMJ boutons under confocal microscopy. Using a pre-embedding immunogold electron microscopy method, we observed that the Dlg protein was widely distributed in the SSR membranes of type Ib boutons (Figures 1F,G) and type Ib boutons (Figure 1H; Gan and Zhang, 2018) but was not present in the ghost boutons (Figure 1I).

### The *dnrx*, *dnlg2*, and *dnlg4* Mutants Had Increased Levels of Ghost Boutons Under TEM

**FIGURE 2:**
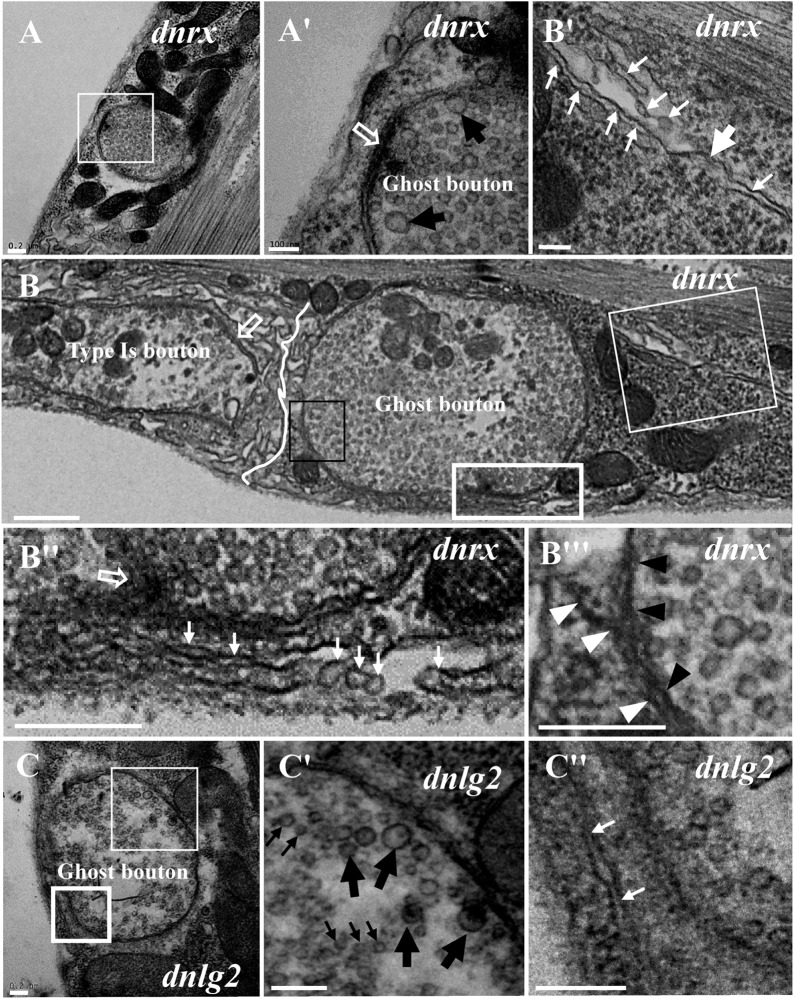
Ultrastructure of the ghost boutons in the *dnrx* and *dnlg2* mutants. In the *dnrx* mutant, ghost boutons have large synaptic vesicles, a synapse and T-bar structure, and no SSR membrane **(A,A′)**. A developing ghost bouton has an obvious boundary with a nearby type Is bouton at one end **(B)** and a T-bar **(B,B″)** and a sparse, thin SSR membrane at the other end **(B,B′,B″)**. The first muscle cell membrane and the axonal membrane are separated in the ghost bouton **(B,B″′)**. In the *dnlg2* mutant, ghost boutons have large synaptic vesicles **(C,C′)** and only one single flat SSR membrane **(C,C″)**. Hollow arrows show T-bars or synapses, large black arrows show large clear vesicles, and small black arrows show small clear vesicles. Thick white arrows show swollen SSR membranes, and thin white arrows show thin SSR membranes. The paired axonal membrane (black wedges) and muscle cell membrane (white wedges) were separated in ghost boutons. **(A′,B′,C′)** Are the enlarged images of the thin white boxes in **(A–C)**, respectively. **(B″,C″)** Are the enlarged images of the thick white boxes in **(B,C)**, respectively. **(B″′)** Is an enlargement of the black box in **(B)**. The white curve shows the boundary between the boutons. Scale bars: **(A,A′,B–C″)**, 200 nm; **(B′)**, 100 nm.

We frequently found ghost boutons in the *dnrx*, *dnlg2*, and *dnlg4* mutants (Table 1). We observed the synapse and T-bar structure in a ghost bouton without an SSR membrane in the *dnrx* mutant (Figures 2A,A′). A long, sparse SSR membrane stretched from the upper (Figures 2B,B′) and lower directions (Figures 2B,B″′), not around the partially naked ghost bouton but in the opposite region of the same ghost bouton that was adjacent to a type Is bouton (Figure 2B). The SSR membranes had an obvious boundary between the ghost bouton and type Is bouton (Figure 2B). The two-layer membranes of the developing ghost boutons, including the inner axon terminal membrane and the outer muscle cell membrane, were separated, and the outer layer participated in the formation of the SSR (Figures 2B,B″′). Moreover, the ghost bouton contained a synapse and T-bar structure with sparse SSR membranes (Figures 2B,B″), which showed that the ghost bouton was developing and forming the SSR.

**TABLE 1:** Ghost boutons and satellite boutons analysis in TEM.

Genotype	WT	*caki*	*dnrx*	*dnlgl*	*dnlg2*	*dnlg3*	*dnlg4*	*OE-dnlg2*	*OE-dnlg3*	*dnlg2;dnlg3*	*dbrat*
Ghost boutons per 6/7muscle	3/15	0/3	5/2	0/3	2/4	0/4	9/3	0/2	0/3	0/2	0/3
Ghost boutons (SEM)	0.2 ± 0.11	0	1.3 ± 0.33	0	1 ± 0.78	0	3 ± 0.58	0	0	0	0
Ghost boutons *P*-value			***		*		***				
Satellite boutons per 6/7muscle	0/15	0/3	0/3	0/3	0/4	0/4	0/3	0/3	19/2	15/2	12/3

^a^All fly strain and analyses are described in section Materials and Methods. ^b^Ghost boutons and Satellite boutons are the total on the muscle 6 and the muscle 7 in segment A3. ^c^P-values for ghost boutons counts were determined by comparison to third instar wild-type (WT). ^d^Using a standard t test analysis. SEM, standard error of the mean. * p < 0.05; *** p < 0.001.

Furthermore, we observed ghost boutons in the *dnlg2* mutant (Figures 2C–C″). The ghost boutons had synaptic vesicles with different diameters (Figures 2C,C′) and a single flat SSR membrane (Figures 2C,C″) or no SSR membrane.

**FIGURE 3:**
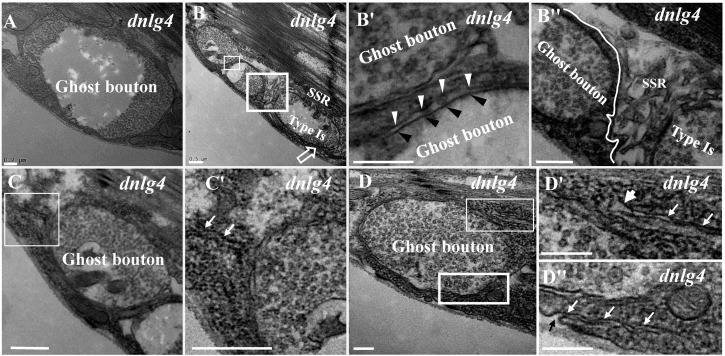
Ultrastructure of the ghost boutons in the *dnlg4* mutant. Ghost boutons have no synapses, T-bar structures, or SSR in the *dnlg4* mutant **(A)**. Beaded ghost boutons in the *dnlg4* mutant **(B)**. No SSR membranes are observable between the ghost boutons **(B,B′)**. The ghost bouton does not share SSR membranes with the adjacent type Is boutons **(B,B″)**. Sparse thin SSR membranes extend to ghost boutons in the *dnlg4* mutant **(C,C′)**. Thin SSR membranes around the ghost bouton from the upper direction **(D,D′)** and downward direction **(D,D″)** in the *dnlg4* mutant. Hollow arrows show T-bars or synapses. Thick white arrows show swollen SSR membranes, and thin white arrows show thin SSR membranes. The axonal membrane (black wedges) is a paired muscle cell membrane (white wedges) in two ghost boutons. Black arrows show the invaginated sites of the SSR membrane. **(B′,C′,D′)** are the enlarged images of the thin white boxes in **(B–D)**, respectively. **(B″,D″)** Are the enlarged images of the thick white boxes in **(B,D)**, respectively. The white curve shows the boundary between the boutons. Scale bars: **(A,B″,D–D″)**, 200 nm; **(B,B′,C,C′)**, 500 nm.

Ghost boutons had synaptic vesicles with different diameters and no synapses, T-bar structures, or SSR membranes (Figure 3A) in the *dnlg4* mutant; the ghost boutons formed bead shapes (Figures 3B,B′) in some NMJ branches without SSR membranes or T-bar structures and resembled normal type Is boutons (Figures 3B,B″) with SSR membranes and T-bar structures. Notably, no SSR membrane was observed between the two ghost boutons, and there were two layers of paired membranes in each ghost bouton (Figures 3B,B′). The inner membrane was from the axon terminal, and the other membrane was from the muscle cell membrane (Figure 3B′). Additionally, the ghost bouton did not share the SSR membrane with the adjacent type Is bouton (Figures 3B,B″). The extremely sparse SSR membranes were near (Figures 3C,C′) or stretched toward and touching (Figure 3D) the ghost boutons from the upper (Figures 3D,3D′) and lower directions (Figures 3D,3D″) but were not wrapped around the ghost bouton. Furthermore, few SSR membranes were swollen (Figure 3D′), and most SSR membranes were thin (Figures 3D′,3D″). The invaginated site was connected to a long thin SSR membrane (Figure 3D″).

**FIGURE 4:**
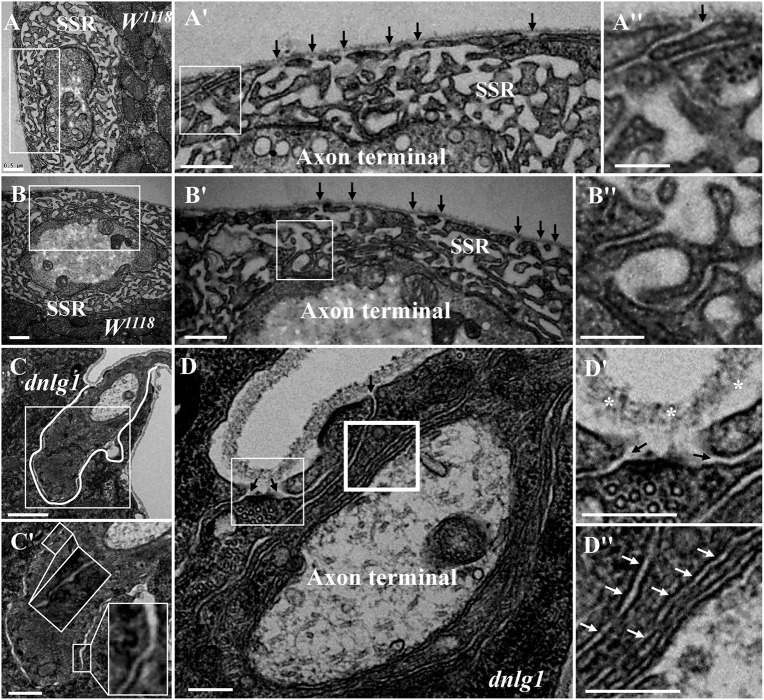
Muscle cell membranes invaginate at multiple sites and form SSR membranes. Six invaginated sites exist outside of the type Is boutons **(A,A′)** in the wild-type strain. An invaginated site with a long SSR membrane **(A″)**. Seven invaginated sites exist outside of the type Ib bouton **(B,B′)** in the wild-type strain. The complex branches of an SSR membrane **(B″)**. The outermost SSR membrane enfolds the axon terminal **(C,C′)** in the *dnlg1* mutant. Three clear and distinct entrances on the cell surface **(D,D′)**. Two layers of SSR membranes around the axon terminal **(D,D″)**. Black arrows show the invaginated sites of the SSR membrane, * Shows the extracellular matrix, and white arrows show thin SSR membranes. **(A′,B′,C′,D′)** Are the enlarged images of the white thin boxes in **(A–D)**, respectively. **(A″,B″)** Are the enlarged images of the white thin boxes in **(A′,B′)**, respectively. **(D″)** Is an enlarged image of the white thick box in **(D)**. The white curve shows the boundary of the bouton. Scale bars, **(A,A′,B,B′,C′)**, 500 nm; **(A″,B″,D–D″)**, 200 nm; **(C)**, 1,000 nm.

Because the extremely sparse SSR membranes stretched toward and touched the ghost boutons from different directions (Figures 3D–D″), we speculated that different invaginated sites were present on the cell membrane near the NMJ bouton. We examined the cell membrane surfaces in the NMJ bouton sections and found that all type Is (Figures 4A–A″) and type Ib (Figures 4B,B′) boutons had several invaginated sites on the cell membrane despite that the invaginated entrance was clear and distinct (Figures 4A′,A″), demonstrating that complex SSR membranes were invaginated by muscle cell membranes at multiple sites. Moreover, these invaginated SSR membranes were repeatedly branched (Figures 4B′,B″) to form the complex SSR of NMJ boutons (Figures 4A,B). Fortunately, we observed an axon terminal that was enwrapped by several layers of SSR membranes in the *dnlg1* mutant (Figures 4C,D). The three sites that adhered to the extracellular matrix were clear and distinct (Figures 4D,D′) and showed several independent clear SSR membranes wrapped around the axon terminal (Figures 4C,C′,D,D″). These data demonstrated the SSR formation process. Initially, the muscle cell membrane invaginates from different sites and extends to a ghost bouton. Then, the ghost bouton is enwrapped with several layers of membranes. Finally, these membranes repeatedly branch and form a complex SSR membrane.

### Identification of the Three Types of Satellite Boutons on TEM

**FIGURE 5:**
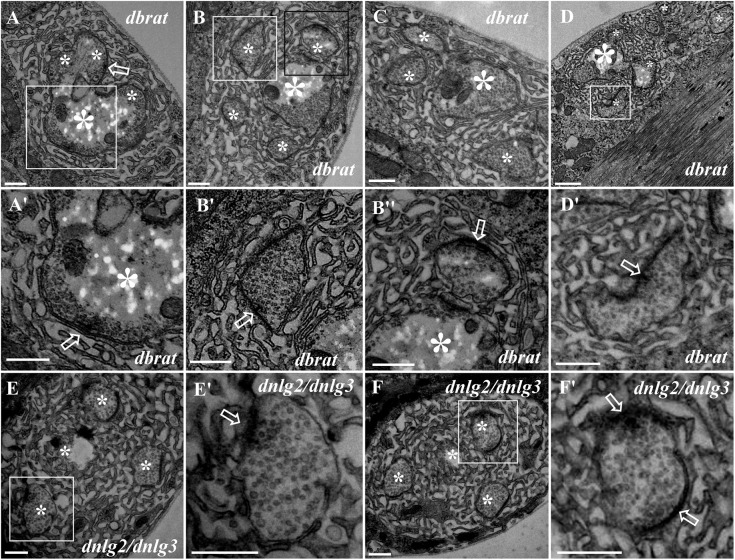
Ultrastructure of the satellite boutons in the *dbrat* and *dnlg2;dnlg3* mutants. Typical satellite boutons in the *dbrat* mutant contain a main bouton and several small boutons **(A–D)**. The main bouton has a typical T-bar **(A′)**. The small boutons have typical T-bars and are filled with dense synaptic vesicles **(B′,B″,D′)**. The atypical satellite boutons in the *dnlg2;dnlg3* double mutant contain only four small boutons **(E,F)** that contain T-bars and are densely loaded with synaptic vesicles **(E′,F′)**. Hollow arrows show T-bars or synapses, large asterisks show the main boutons, and small asterisks show small boutons or branches. **(A′,B′,D′,E′,F′)** Are the enlarged images of the white boxes in **(A,B,D–F)**, respectively. **(B″)** Is an enlargement of the black box in **(B)**. Scale bars: **(A–C,E–F′,D′)**, 500 nm; **(D)**, 1,000 nm.

Because we previously demonstrated by confocal laser scanning microscopy that *dbrat* mutants had numerous satellite boutons (Shi et al., 2013), we used a *dbrat* mutant to identify the ultrastructural features of satellite boutons as a positive control. Under TEM, satellite boutons had typical and atypical forms. Typical satellite boutons consisted of a large main bouton and several small boutons observable in serial sections (Figures 5A–D); the main bouton was in the center, and the small boutons, most less than 500 nm in diameter and appearing as several “satellites,” were spread around the main bouton in the *dbrat* mutant (Figures 5B–D). The synaptic vesicles were distributed around the main bouton and were absent in the center of the main bouton (Figures 5A,B), similar to the normal type I bouton in wild-type flies (Figure 1A). The small satellite boutons were completely filled with synaptic vesicles (Figures 5B′,B″,D′), unlike the main bouton (Figures 5A,B). Synapses and typical T-bar structures were observed in the main bouton (Figures 5A,A′) and in the satellite boutons (Figures 5B′,B″,D′).

**FIGURE 6:**
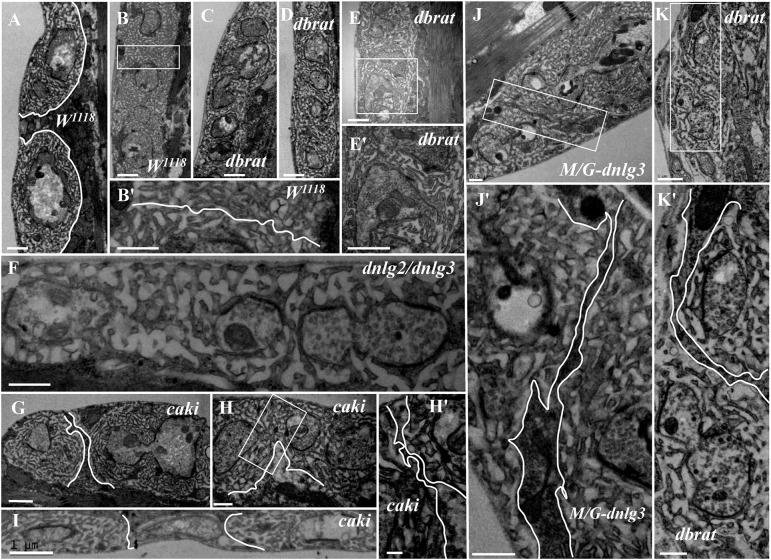
Ultrastructural comparison of small boutons and atypical satellite boutons. Both adjacent type Ib boutons in the wild-type strain have independent SSR membranes **(A–B′)**. The atypical satellite boutons contain several beaded small boutons, and they are wrapped in the same SSR membrane in the *dbrat* mutant **(C–E′)** and in the *dnlg2;dnlg3* double mutant **(F)**. The small beaded boutons have obvious boundaries and orientations between the small-sized boutons in the *caki* mutant **(G–H′)** and in the rescued *MHC-Gal4;UAS-dnlg3* (*M/G-dnlg3*) strain **(J,J′)** and the *dbrat* mutant **(K,K′)**. **(B′,E′,H′,J′,K′)** Are enlarged images of the white boxes in **(B,E,H,J,K)**, respectively. The white curve shows the boundary between the boutons. Scale bars: **(A–C,E,E′,F,I,K)**, 1,000 nm; **(B′,D,G,J,J′,H,K′)**, 500 nm; **(H′)**, 200 nm.

In contrast to the typical satellite boutons, two types of atypical satellite boutons were observed. In the first type of atypical satellite bouton, the central main bouton was absent or small, and several small boutons were grouped together (Figures 5E,F) with synapses (Figures 5E′,F′), as observed in the *dnlg2;dnlg3* double mutant. In the other atypical satellite boutons, there was no central main bouton, and the small irregular boutons were arranged in a bead-like shape in three *dbrat* mutants (Figures 6C–E′) and in the *dnlg2;dnlg3* double mutant (Figure 6F), showing a common SSR membrane. In contrast, in wild-type flies, the adjacent type Ib boutons were large and markedly spherical or ellipsoidal, with clear boundaries comprising the SSR membranes (Figure 6A). The small beaded boutons were occasionally visible in an interconnected SSR in the wild-type strain (Figure 6B), and a relatively obvious boundary (Figure 6B′) was observable between the bead boutons. The small beaded boutons could not be identified as satellite boutons (Figures 6G–K) without a common SSR membrane by TEM, and even the small NMJ boutons were distributed in beads in the *caki* mutant under confocal microscopy (Sun et al., 2009). Obvious differences in the boundaries and orientation between the different small-sized boutons in the *caki* mutant (Figures 6G–I) were observed by TEM. Satellite boutons could be observed in the *Elav-Gal4;UAS-dnlg3* strain and in the *dbrat* mutant (Table 1), but there were still notable boundaries between the small NMJ boutons; thus, these boutons could not be considered satellite boutons because no common SSR was shared (Figures 8J,J′,K,K′).

### DNlg2 and DNlg3 Synergistically Led to Satellite Boutons as Determined by TEM

**FIGURE 7:**
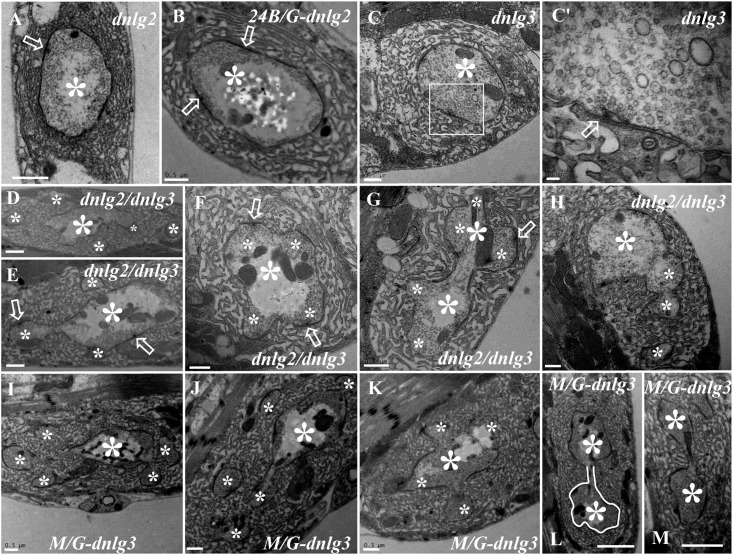
DNlg2 and DNlg3 synergistically regulate satellite boutons on transmission electron microscopy (TEM). No satellite boutons in the *dnlg2* mutant **(A)**, the rescued *24B-Gal4;UAS-dnlg2* strain **(B)**, and the *dnlg3* **(C,C′)** mutant. The satellite boutons in the *dnlg2;dnlg3* double mutant **(D–E)**. The cruciform bouton **(F)**, exogenous small boutons **(G)**, and repeatedly growing small boutons from a large neuromuscular junction (NMJ) bouton **(H)**. Satellite boutons around the main bouton in the rescued *MHC-Gal4;UAS-dnlg3* strain **(I,J)**. More cruciform boutons **(K)**. The large irregular boutons in a common SSR membrane **(L,M)**. Hollow arrows show T-bars and synapses, large asterisks show the main boutons, and small asterisks show small boutons or branches. The white curve shows an irregular bouton shape. Scale bars: **(A–C,D–K)**, 500 nm; **(C′)**, 100 nm; **(L,M)**, 1,000 nm.

We did not observe satellite boutons in the *dnlg2* (Figure 7A) mutants or rescued *24B-Gal4;UAS-dnlg3* (Figure 7B) and *dnlg3* (Figures 7C,C′) mutants as reported by Sun et al. (2011) and Xing et al. (2018). However, we frequently observed typical satellite boutons in the *dnlg2;dnlg3* double mutant (Figures 7D,E and Table 1). All main and small boutons shared a common SSR membrane that was fairly regular and layered, but the shape appeared to be extremely irregular in the large main boutons and relatively small main boutons, including cruciform boutons (Figure 7F), exogenous small boutons (Figure 7G), and small boutons repeatedly growing from a large NMJ bouton (Figure 7H). Interestingly, more prominent typical satellite boutons were observed in the rescued *MHC-Gal4;UAS-dnlg3* strain. We observed that five small boutons were distributed around the main bouton (Figures 7I,J) and the cruciform bouton (Figure 7K). The large irregular boutons had a common SSR (Figures 7L,M), whereas the type Ib boutons were mostly spherical or ellipsoidal in wild-type flies (Figures 1A, 6A).

### The *dnrx* and *dnlgs* Jointly Maintained the Balance of Ghost and Satellite Boutons as Determined by Confocal Microscopy

**FIGURE 8:**
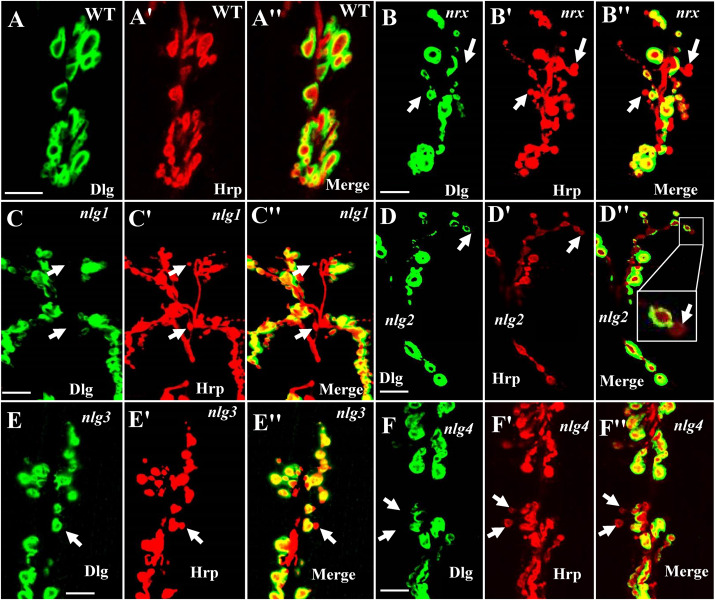
Ghost boutons in the *dnrx* mutant and 4 *dnlg* mutants. Almost no ghost boutons were found in wild-type flies **(A–A″)**. Ghost boutons in the *dnrx* **(B–B″)**, *dnlg1* **(C–C″)**, *dnlg2* **(D–D″)**, *dnlg3* **(E–E″)**, and *dnlg4* **(F–F″)** mutants. Dlg is a marker for postsynaptic SSR, and Hrp is a marker for the presynaptic membrane. The thick white arrows show the ghost boutons that have no Dlg signals. Scale bars, 10 μm.

**FIGURE 9:**
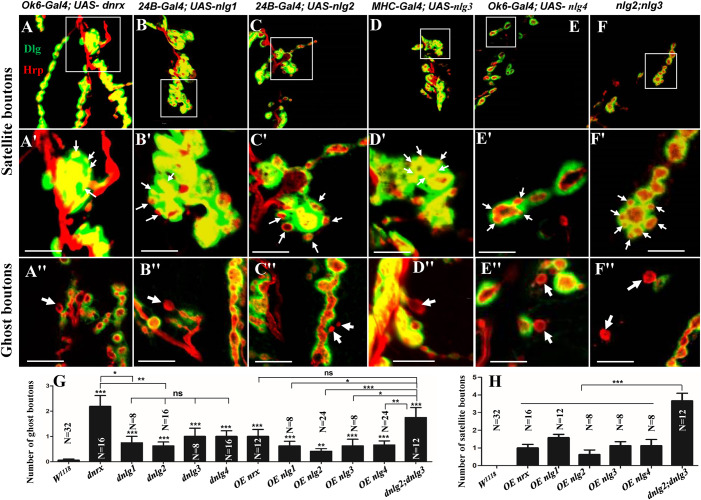
Satellite and ghost boutons in associated strains of *dnrx* and *dnlg*s. Satellite boutons in overexpression strains of *dnrx* **(A,A′)**, *dnlg1* **(B,B′)**, *dnlg2* **(C,C′)**, *dnlg3* **(D,D′)**, and *dnlg4* **(E,E′)**. Satellite boutons in the *dnlg2;dnlg3* double mutant **(F,F′)**. Quantity statistics of ghost boutons **(G)** and satellite boutons **(H)**. OE, overexpression. **(A′,B′,C′,D′,E′,F′)** Are enlarged images of the white boxes in **(A–F)**, respectively. The thick white arrows show the ghost boutons, and the thin white arrows show the satellite boutons. *P < 0.05; **P < 0.01; ***P < 0.001; ns, no significance. Scale bars, 10 μm.

Our TEM data showed the ultrastructures of the ghost and satellite boutons in the *dnrx*, *dnlg2*, and *dnlg4* mutants. The ghost and satellite boutons were observed under confocal microscopy with a large field and three-dimensional view, utilizing immunofluorescence labeling of Dlg, a marker for the postsynaptic SSR, and Hrp, a marker for the presynaptic membrane. To confirm whether the *dnlg1* and *dnlg3* mutants exhibited ghost boutons and whether the *dnrx-* and *dnlg*-overexpressing strains had satellite synapses, we analyzed the numbers of ghost and satellite boutons in the *dnrx* and *dnlg* mutants, *dnrx-* and *dnlg-*overexpressing strains, and the associated double mutants by confocal microscopy (Figures 8, 9).

Consistent with the electron microscopy results, we rarely observed ghost boutons in the *W*^1118^ strain (0.063 ± 0.043, *N* = 32) (Figures 8A–A″) and frequently observed ghost boutons in the *dnrx* (2.188 ± 0.430, *N* = 16) (Figures 8B–B″), *dnlg1* (0.750 ± 0.250, *N* = 8) (Figures 8C–C″), *dnlg2* (0.625 ± 0.154, *N* = 16) (Figures 8D–D″), *dnlg3* (1.00 ± 0.327, *N* = 8) (Figures 8E–E″), and *dnlg4* (1.00 ± 0.224, *N* = 16) (Figures 8F–F″) mutants (Figure 9G and Table 2). The *dnrx* mutant had the most ghost boutons, exhibiting significantly more than that the *dnlg1* (*P* = 0.035) and *dnlg2* (*P* = 0.0018) mutants (Figure 9G). The number of ghost boutons was not significantly different among the four *dnlg* mutants (Figure 9G).

**TABLE 2:** Ghost boutons and satellite boutons analysis in confocal microscopy.

Genotype	WT	*dnrx*	*dnlgl*	*dnlg2*	*dnlg3*	*dnlg4*	*OE-dnrx*	*OE-dnlgl*	*OE-dnlg2*	*OE-dnlg3*	*OE-dnlg4*	*dnlg2;dnlg3*
Ghost boutons per 6/7muscles	2/32	35/16	6/8	10/16	8/8	16/16	12/12	5/8	10/24	5/8	16/24	21/12
Ghost boutons (SEM)	0.06 ± 0.04	2.19 ± 0.43	0.75 ± 0.25	0.63 ± 0.15	1 ± 0.33	1 ± 0.22	1 ± 0.28	0.63 ± 0.18	0.42 ± 0.10	0.63 ± 0.26	0.67 ± 0.16	1.75 ± 0.39
Ghost boutons *P*-value		***	***	***	***	***	***	***	**	***	***	***
Satellite boutons per 6/7muscles	0/32	0/16	0/8	0/16	0/8	0/16	16/16	19/12	5/8	9/8	9/8	44/12

^a^All fly strain and analyses are described in section Materials and Methods. ^b^Ghost boutons and Satellite boutons are the total on the muscle 6 and the muscle 7 in segment A2 and A3. ^c^P-values for ghost boutons counts were determined by comparison to third instar wild-type (WT). ^d^Using a standard t-test analysis. SEM, standard error of the mean. ** 0.05 < p < 0.01; *** p < 0.001.

No satellite boutons were observed in the wild-type strain or in the *dnrx*, *dnlg1*, *dnlg2*, *dnlg3*, and *dnlg4* mutants (data not shown), but satellite boutons were frequently observed in all overexpression strains of *dnrx* (1.000 ± 0.204, *N* = 16) (Figures 9A,A′), *dnlg1* (1.583 ± 0.193, *N* = 12) (Figures 9B,B′), *dnlg2* (0.625 ± 0.263, *N* = 8) (Figures 9C,C′), *dnlg3* (1.125 ± 0.227, *N* = 8) (Figures 9D,D′), and *dnlg4* (1.125 ± 0.350, *N* = 8) (Figures 9E,E′, 9H and Table 2). More satellite boutons were observed in the *24B-Gal4;UAS-dnlg3* strain (data not shown) than in the *MHC-Gal4;UAS-dnlg3* strain (Figures 9D, D′), but the difference between the two strains was not significant. Consistent with the electron microscopy results, satellite boutons (3.667 ± 0.432, *N* = 12) frequently appeared in the *dnlg2;dnlg3* double mutant (Figures 9F,F′,G and Table 2), and up to six small boutons surrounded the main bouton (Figures 9F,F′).

Interestingly, ghost boutons frequently appeared in the *dnlg2;dnlg3* double mutant (1.750 ± 0.392, *N* = 12) (Figure 9F″) and in the strains overexpressing *dnrx* (1.000 ± 0.275, *N* = 12) (Figure 9A″), *dnlg1* (0.625 ± 0.193, *N* = 8) (Figure 9B″), *dnlg2* (0.416 ± 0.102, *N* = 24) (Figure 9C″), *dnlg3* (0.625 ± 0.263, *N* = 8) (Figure 9D″), and *dnlg4* (0.667 ± 0.156, *N* = 24) (Figure 9E″). The number of satellite boutons in the *dnlg2;dnlg3* double mutant was significantly higher than that in the other overexpression strains (Figure 9H), and the number of ghost boutons was significantly higher than those of the five overexpression strains (Figure 9G).

These findings indicated that appropriate doses of *dnrx* and *dnlgs* maintained the normal development of the synapse. The *dnrx* and 4 *dnlg* mutations led to ghost boutons that were markers of poor synaptic development, and the overexpression of *dnrx* and the four *dnlg*s led to satellite boutons that were markers of excessive synaptic development. Excessive synaptic development could also lead to ghost boutons.

## Discussion

### The Characteristics and Growth of Ghost Boutons on TEM

Ghost boutons are undifferentiated and immature synaptic boutons that contain synaptic vesicles and lack active zones and postsynaptic structures, although these structures can differentiate into mature boutons over prolonged periods (Ataman et al., 2008) and do exist in wild-type organisms at a very low frequency, as observed with confocal microscopy (Ataman et al., 2006; Loya et al., 2014) but not with TEM. Ghost bouton budding was observable from both type Ib and type Is boutons (Piccioli and Littleton, 2014) under confocal microscopy. Ghost boutons are regulated by bone morphogenetic protein (BMP) (Piccioli and Littleton, 2014), Wnt signaling molecules (Packard et al., 2002; Ataman et al., 2008), and PFN1 mutants (Wu et al., 2017).

Ultrastructural evidence also confirmed that ghost boutons were poorly developed in the NMJ bouton. Under TEM, ghost boutons lacked active zones, SSR membranes, and mitochondria and had dense clear synaptic vesicles (Packard et al., 2002). Furthermore, we found that ghost boutons contained synaptic vesicles with multiple diameters in wild-type flies and mutant strains; the large synaptic vesicles were similar to those in the immature axonal neurites of the *Drosophila* larval ventral nerve cord (Gan et al., 2014), while the large vesicles almost completely disappeared in both NMJ type Ib boutons (Jia et al., 1993) and mature axonal neurites (Gan et al., 2014). Ghost boutons contained very few signals for Brp (Ataman et al., 2006, 2008), a marker of synaptic active zones and T-bar structures. Early NMJ type Ib boutons in *Drosophila* embryos had frequent T-bar structures and mitochondria but no SSR membrane (Prokop et al., 1996; Koper et al., 2012). We observed few T-bar structures in the ghost boutons in the *dnrx* mutant, for which the presynaptic structure could be independently formed without the induction of a postsynaptic structure (Prokop et al., 1996). Furthermore, ghost boutons could be differentiated into mature boutons with a dense SSR over prolonged periods (Ataman et al., 2008), and we observed sparse linear SSR membranes that developed the SSR in wild-type flies and in the *dnrx*, *dnlg2*, and *dnlg4* mutants.

The SSR membrane originated from the muscle cell membrane, stretched to the ghost bouton, and enfolded and branched into the axon terminal. We observed clear and obvious sites in wild-type (Figures 4A′,B′) and *dnrx* (Figure 3D″) and *dnlg1* mutant (Figures 4D,D′) flies, directly indicating that the SSR membranes of the NMJ bouton originated from different sites of the muscle cell membrane and developed into complicated SSR membranes from multiple sources. Furthermore, the invaginated sites formed different layers of the SSR membranes; the outer layer of the SSR membrane from the outer invaginated sites formed the outer layers of the SSR membrane in the *dnlg1* mutant (Figures 4C–D′), while the innermost invaginated sites formed synapses. Then, the single SSR membrane was repeatedly branched (Figures 4A″,B″) and formed the complex SSR of the NMJ bouton.

The developing ghost bouton had a relatively independent SSR membrane. In the *dnrx* and *dnlg4* mutants, the sparse SSR membranes stretched toward and touched the ghost boutons from different directions, and these membranes could not generate the SSR membrane of the other bouton. Furthermore, the ghost bouton might have been adjacent to a type Is bouton, but there was a clear boundary between the ghost bouton and the type Is bouton (Figure 3B″) in the *dnlg4* mutant. Moreover, the ghost boutons contained synapse and T-bar structures (Figure 2A) with sparse SSR membranes in the other directions (Figures 4D–D″).

### Mutations in *dnrx* and *dnlg*s Lead to Ghost Boutons

The SSR membrane was swollen near the ghost boutons (Figure 1E′) in wild-type flies and was thin in the *dnrx* (Figure 2B′), *dnlg2* (Figure 2C″), and *dnlg4* (Figures 3D′,D″) mutants, which frequently showed ghost boutons. Therefore, the swollen SSR membrane had components from the muscle cell membrane, which could contribute to normal SSR membrane branching and development; a reduction in or the absence of the swollen SSR membrane could result in ghost boutons that did not have SSR membranes or with thin SSR membranes in type Ib boutons in the *dnlg2* mutant (Sun et al., 2011) or with a small SSR area in type Ib boutons in the *dnlg4* mutant (Li et al., 2013; Zhang et al., 2017). In fact, the SSR thickness was reduced in the *dnrx* mutant (data not shown).

Neurexins and neuroligins are synaptic adhesion molecules commonly associated with autism and schizophrenia (Zhang et al., 2018) and are reportedly involved in synapse formation and synaptic transmission in *Drosophila* NMJ type Ib boutons (Sun et al., 2009, 2011). Although we prepared serial slices, we did not observe typical ghost boutons in the *dnlg1* and *dnlg3* mutants by TEM, possibly due to occlusion of the grids. Therefore, we analyzed the ghost bouton numbers by confocal microscopy with a large field and three-dimensional view. We confirmed that ghost boutons were regulated by the five synaptic adhesion molecules, DNrx, DNlg1, DNlg2, DNlg3, and DNlg4, in this study. Interestingly, all the five related mutants had defects in synaptic morphology and function in NMJ, which could lead to ghost boutons, the poorly developed boutons.

Ghost boutons are also regulated by the BMP (Piccioli and Littleton, 2014) and Wnt signaling molecules (Ataman et al., 2008; Packard et al., 2002) and by PFN1 (Wu et al., 2017), and these molecules were involved in regulation of the cytoskeletal network. DNrx, DNlg1, and DNlg4 regulated synaptic growth via the BMP signaling pathway at the *Drosophila* NMJ (Banerjee et al., 2017; Zhang et al., 2017), and DNrx, DNlg1, DNlg2, and DNlg3 regulated postsynaptic actin filament (Xing et al., 2018), a key cytoskeletal component in NMJ, which demonstrated that DNrx and DNlgs mediated synaptic cytoskeleton disorder and caused ghost boutons.

Although DNrx was generally distributed in the presynaptic membrane, DNlgs were distributed in the postsynaptic membrane, and mutations in *dnrx*, *dnlg1*, *dnlg2*, *dnlg3*, and *dnlg4* led to the frequent occurrence of ghost boutons, which suggested that poorly developed boutons or bouton-related genetic changes might be associated with autism. Therefore, with respect to autism caused by DNrx and DNlgs, attention should be paid to not only their associations with impaired synaptic transmission but also their induction of ghost boutons, the immature synaptic boutons.

### The Characteristics of Satellite Boutons on TEM

Satellite boutons are described as parent boutons of normal sizes with many attached small boutons (Menon et al., 2013), and these structures bud from axonal segments connecting two adjacent boutons on TEM (Torroja et al., 1999). In this study, we characterized three subtypes of satellite boutons with TEM. The typical satellite bouton comprises a large main bouton and several small boutons, with the main bouton in the center and the small boutons, appearing as several satellites, budding around the main bouton. If the main bouton or parent bouton was also small, only a few small boutons were grouped together, which could be considered an atypical satellite bouton with a shared SSR membrane (Figures 7E,F). Based on confocal microscopy, the small NMJ boutons were shown to be distributed as beads in both the *caki* (Sun et al., 2009) and *dbrat* (Shi et al., 2013) mutants, and distinguishing between the small-sized boutons and small satellite boutons was difficult. In TEM, the small, beaded NMJ boutons shared SSR membranes in the *dbrat* mutant and the *dnlg2;dnlg3* mutant (Figure 6), and we believed that the small boutons represented the other atypical satellite bouton variety. In the *caki, dbrat*, and *dnlg2;dnlg3* mutants, the small beaded boutons were simple abnormally small boutons with obvious boundaries and orientations (Figure 6). Therefore, the shared SSR membrane among the NMJ boutons was used to identify satellite boutons on TEM.

### Overexpressing DNrx and DNlgs Leads to Satellite Boutons

In contrast to the mutations of *dnrx* and *dnlgs* leading to ghost boutons, satellite boutons were observed in all five strains overexpressing *dnrx* and *dnlgs*. The satellite boutons were promoted by DNlg4 overexpression and required BMP signaling (Zhang et al., 2017). Since DNrx and DNlg1 also regulated synaptic growth via the BMP signaling pathway (Banerjee et al., 2017), the satellite boutons in the strains of DNrx and DNlg1 overexpression were likely to be regulated via the BMP signaling pathway. No satellite boutons were observed in the rescued *24B-Gal4;UAS-nlg2* strain, but satellite boutons frequently appeared in the *24B-Gal4;UAS-nlg2* overexpression strain, demonstrating that an appropriate dose of DNlg2 regulated the overgrowth of synaptic boutons. Moreover, DNlg4 also had a dosage-sensitive genetic interaction with the components of the BMP pathway (Zhang et al., 2017). No satellite boutons were observed in the *dnrx*, *dnlg1*, *dnlg2*, dnlg*3*, and *dnlg4* mutants. However, satellite boutons frequently appeared in the *dnlg2;dnlg3* double mutants, which suggested that the postsynaptic adhesion molecules *dnlg2* and *dnlg3* synergistically regulated satellite boutons that could be considered an indicator of synaptic overgrowth.

### DNrx and DNlgs Regulate the Balance of Ghost and Satellite Boutons

Neurexin and neuroligins are well-known synaptic adhesion molecules associated with autism, and they have close evolutionary homologs in invertebrates and vertebrates. There are three *nrx* genes in mammalians but only one neurexin gene in *Drosphila*. There are four nlg genes in rodents, which encode Nlg1, Nlg2, Nlg3, and Nlg4, while five *nlg* genes, *nlg1*, *nlg2*, *nlg3*, *nlg4*, and *nlg4y*, in the human genome. The *nlg4y* was similar in structure to that which encodes Nlg4Y on the Y chromosome. However, the four *dnlg* genes, encoding DNlg1–4, were not one-to-one homologous to mammalian Nlg1–4, and they were similar to human Nlg1 in homology, which showed that the four *dnlg* genes were fairly convergent on specific phenotypes, such as satellite boutons and ghost bouton.

**FIGURE 10:**
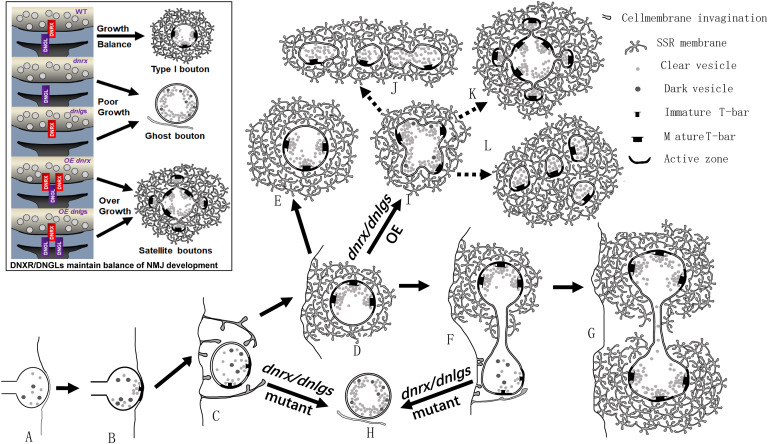
*dnrx* and *dnlg*s regulate the balance of ghost boutons and satellite boutons. **(A–L)** show the process by which *dnrx* and *dnlg*s regulate the balance of ghost and satellite boutons. The newborn NMJ bouton contains vesicles with different diameters and contacts the surfaces of muscle cells **(A)**. Immature synaptic connection at the contact site **(B)**. The plasma membrane shows invagination at multiple sites and begins to branch **(C)**. Developing NMJ bouton with immature synapses and few vesicles with different diameters **(D)**. Mature NMJ bouton with mature synapses and dense clear synaptic vesicles **(E)**. The newborn NMJ bouton buds from the existing bouton **(F)** and develops two mature NMJ boutons **(G)** (Figure 6A). With mutations in *dnrx* and *dnlg*s, the newborn boutons form ghost boutons with dense vesicles with different diameters and spare the SSR membrane and synapse **(H)**. With overexpression of *dnrx* and *dnlg*s, the NMJ bouton overgrows into satellite boutons **(I)**, and the satellite boutons form three types, including typical satellite boutons **(K)**, atypical satellite boutons with beaded small boutons **(J)**, and atypical satellite boutons with gathered small boutons **(L)**. The black box shows that *dnrx* and *dnlg*s regulate the balance of ghost and satellite boutons: mutation in *dnrx* and *dnlg*s leads to ghost boutons, which indicate poor synaptic growth, and overexpression in *dnrx* and *dnlg*s leads to satellite boutons, which indicate synapse overgrowth.

All mutants of *dnrx* and *dnlgs* had abnormal presynaptic and postsynaptic phenotypes. *dnrx* and *dnlg4* mainly played presynaptic roles (Li et al., 2007; Zhang et al., 2017), *dnlg1* and *dnlg3* mainly played postsynaptic functions (Banovic et al., 2010; Xing et al., 2018), and *dnlg2* played pre- and postsynaptic roles (Sun et al., 2011; Chen et al., 2012). Therefore, *dnrx* and *dnlgs* regulated the presynaptic and postsynaptic cytoskeletal networks through their extracellular domains and jointly maintained the development of NMJ boutons in *Drosophila*. For a single NMJ bouton, *dnrx*, *dnlg1*, *dnlg2*, *dnlg3*, and *dnlg4* mutations led to ghost boutons that indicate poor development in the NMJ bouton. The overexpression of *dnrx* and the four *dnlg*s and the *dnlg2*/*dnlg3* double mutation led to satellite boutons that could indicate overgrowth. These findings suggested that both DNrx and DNlgs jointly maintained the balance of poor development and overgrowth in NMJ boutons by regulating ghost and satellite boutons (Figure 10 black box).

Based on the above data, we described the pattern of ghost and satellite boutons. The vacuolar axon terminal with vesicles of different diameters reached and contacted the surfaces of muscle cells (Figure 10A; Koper et al., 2012; Gan et al., 2014), and the vesicles gradually underwent synaptic vesicle docking and immature T-bar assembly (Figure 10B; Koper et al., 2012; Gan et al., 2014). The axon terminal invaginated into the muscle cell and formed double-layer membranes with the cell membrane (Figure 10C). Then, the other plasma membrane invaginated and enfolded the axon terminal from multiple sites (Figure 10D), and the SSR membrane further branched and folded (Jia et al., 1993), finally developing into mature NMJ boutons with mature T-bar structures and dense synaptic vesicles (Figure 10E; Jia et al., 1993; Gan et al., 2014). The newborn bouton without an SSR was derived (Figure 10F) from the existing NMJ bouton during development (Zito et al., 1999) and developed into another mature NMJ bouton (Figure 10G). Once the developing NMJ bouton (Figure 10C) or the newborn bouton (Figure 10F) was disturbed, ghost boutons with vesicles of different diameters formed and spared the SSR membrane and synapse (Figure 10H). The number of ghost boutons was regulated by DNrx and the four DNlgs as well as by DNT1, DNT2, Spz (Sutcliffe et al., 2013), LIM kinase (Piccioli and Littleton, 2014), *PFN1* (Wu et al., 2017), and physicochemical factors (Ataman et al., 2008).

If the NMJ boutons developed with a common SSR membrane, they would have budded like yeast (Figure 10I; Lee and Wu, 2010) and developed into mature satellite boutons (Lee and Wu, 2010). The mature satellite boutons had a typical appearance with small surrounding boutons (Figure 10K) and two atypical morphologies with small beaded boutons (Figure 10J) and small gathered boutons (Figure 10L).

## Data Availability Statement

All datasets generated for this study are included in the article/supplementary material.

## Author Contributions

All authors had full access to all data in the study and take responsibility for the integrity of the data and the accuracy of the data analysis. GG and XW: study concept and design. GG, GJ, and MY: acquisition of the data, analysis and interpretation of the data. GG and ZC: drafting of the manuscript, critical revision of the manuscript for important intellectual content, administrative, technical, and material support. GG and MY: statistical analysis. GG: funding and study supervision.

## Conflict of Interest

The authors declare that the research was conducted in the absence of any commercial or financial relationships that could be construed as a potential conflict of interest.

## References

[B1] Ataman B. Ashley J. Gorczyca M. Ramachandran P. Fouquet W. Sigrist S. J. (2008). Rapid activity-dependent modifications in synaptic structure and function require bidirectional Wnt signaling. *Neuron* 57 705–718. 10.1016/j.neuron.2008.01.026 18341991 PMC2435264

[B2] Ataman B. Budnik V. Thomas U. (2006). Scaffolding proteins at the Drosophila neuromuscular junction. *Int. Rev. Neurobiol.* 75 181–216. 10.1016/S0074-7742(06)75009-7 17137929

[B3] Atwood H. L. Govind C. K. Wu C. F. (1993). Differential ultrastructure of synaptic terminals on ventral longitudinal abdominal muscles in Drosophila larvae. *J. Neurobiol.* 24 1008–1024. 10.1002/neu.480240803 8409966

[B4] Banerjee S. Venkatesan A. Bhat M. A. (2017). Neurexin, Neuroligin and Wishful Thinking coordinate synaptic cytoarchitecture and growth at neuromuscular junctions. *Mol. Cell Neurosci.* 78 9–24. 10.1016/j.mcn.2016.11.004 27838296 PMC5219945

[B5] Banovic D. Khorramshahi O. Owald D. Wichmann C. Riedt T. Fouquet W. (2010). Drosophila neuroligin 1 promotes growth and postsynaptic differentiation at glutamatergic neuromuscular junctions. *Neuron* 66 724–738. 10.1016/j.neuron.2010.05.020 20547130

[B6] Chai A. Withers J. Koh Y. H. Parry K. Bao H. Zhang B. (2008). hVAPB, the causative gene of a heterogeneous group of motor neuron diseases in humans, is functionally interchangeable with its Drosophila homologue DVAP-33A at the neuromuscular junction. *Hum. Mol. Genet.* 17 266–280. 10.1093/hmg/ddm303 17947296 PMC3516386

[B7] Chen Y. C. Lin Y. Q. Banerjee S. Venken K. Li J. Ismat A. (2012). Drosophila neuroligin 2 is required presynaptically and postsynaptically for proper synaptic differentiation and synaptic transmission. *J. Neurosci.* 32 16018–16030. 10.1523/JNEUROSCI.1685-12.2012 23136438 PMC3508708

[B8] Dickman D. K. Lu Z. Meinertzhagen I. A. Schwarz T. L. (2006). Altered synaptic development and active zone spacing in endocytosis mutants. *Curr. Biol.* 16 591–598. 10.1016/j.cub.2006.02.058 16546084

[B9] Endris V. Wogatzky B. Leimer U. Bartsch D. Zatyka M. Latif F. (2002). The novel Rho-GTPase activating gene MEGAP/srGAP3 has a putative role in severe mental retardation. *Proc. Natl. Acad. Sci. U.S.A.* 99 11754–11759. 10.1073/pnas.162241099 12195014 PMC129341

[B10] Fuentes-Medel Y. Logan M. A. Ashley J. Ataman B. Budnik V. Freeman M. R. (2009). Glia and muscle sculpt neuromuscular arbors by engulfing destabilized synaptic boutons and shed presynaptic debris. *PLoS Biol.* 7:e1000184. 10.1371/journal.pbio.1000184 19707574 PMC2724735

[B11] Gan G. Lv H. Xie W. (2014). Morphological identification and development of neurite in Drosophila ventral nerve cord neuropil. *PLoS One* 9:e105497. 10.1371/journal.pone.0105497 25166897 PMC4148333

[B12] Gan G. Zhang C. (2018). The precise subcellular localization of Dlg in the Drosophila larva body wall using improved pre-embedding immuno-EM. *J. Neurosci. Res.* 96 467–480. 10.1002/jnr.24139 29231975

[B13] Jia X. X. Gorczyca M. Budnik V. (1993). Ultrastructure of neuromuscular junctions in Drosophila: comparison of wild type and mutants with increased excitability. *J. Neurobiol.* 24 1025–1044. 8409967 10.1002/neu.480240804 PMC4664446

[B14] Koper A. Schenck A. Prokop A. (2012). Analysis of adhesion molecules and basement membrane contributions to synaptic adhesion at the Drosophila embryonic NMJ. *PLoS One* 7:e36339. 10.1371/journal.pone.0036339 22558441 PMC3340374

[B15] Larkin A. Chen M. Y. Kirszenblat L. Reinhard J. van Swinderen B. Claudianos C. (2015). Neurexin-1 regulates sleep and synaptic plasticity in Drosophila melanogaster. *Eur. J. Neurosci.* 42 2455–2466. 10.1111/ejn.13023 26201245

[B16] Lee J. Wu C. F. (2010). Orchestration of stepwise synaptic growth by K+ and Ca2+ channels in Drosophila. *J. Neurosci.* 30 15821–15833. 10.1523/JNEUROSCI.3448-10.2010 21106821 PMC3075884

[B17] Li J. Ashley J. Budnik V. Bhat M. A. (2007). Crucial role of Drosophila neurexin in proper active zone apposition to postsynaptic densities, synaptic growth, and synaptic transmission. *Neuron* 55 741–755. 17785181 10.1016/j.neuron.2007.08.002 PMC2039911

[B18] Li W. Yao A. Zhi H. Kaur K. Zhu Y. C. Jia M. (2016). Angelman syndrome protein Ube3a regulates synaptic growth and endocytosis by inhibiting BMP signaling in Drosophila. *PLoS Genet.* 12:e1006062. 10.1371/journal.pgen.1006062 27232889 PMC4883773

[B19] Li Y. Zhou Z. Zhang X. Tong H. Li P. Zhang Z. C. (2013). Drosophila neuroligin 4 regulates sleep through modulating GABA transmission. *J. Neurosci.* 33 15545–15554. 10.1523/JNEUROSCI.0819-13.2013 24068821 PMC6618453

[B20] Loya C. M. McNeill E. M. Bao H. Zhang B. Van Vactor D. (2014). miR-8 controls synapse structure by repression of the actin regulator enabled. *Development* 141 1864–1874. 10.1242/dev.105791 24718988 PMC3994775

[B21] Menon K. P. Carrillo R. A. Zinn K. (2013). Development and plasticity of the Drosophila larval neuromuscular junction. *Wiley Interdiscip. Rev. Dev. Biol.* 2 647–670. 10.1002/wdev.108 24014452 PMC3767937

[B22] Miller D. L. Ballard S. L. Ganetzky B. (2012). Analysis of synaptic growth and function in Drosophila with an extended larval stage. *J. Neurosci.* 32 13776–13786. 10.1523/JNEUROSCI.0508-12.2012 23035089 PMC3482831

[B23] Monastirioti M. Gorczyca M. Rapus J. Eckert M. White K. Budnik V. (1995). Octopamine immunoreactivity in the fruit fly *Drosophila melanogaster*. *J. Comp. Neurol.* 356 275–287. 7629319 10.1002/cne.903560210 PMC4664080

[B24] Packard M. Koo E. S. Gorczyca M. Sharpe J. Cumberledge S. Budnik V. (2002). The Drosophila Wnt, wingless, provides an essential signal for pre- and postsynaptic differentiation. *Cell* 111 319–330. 12419243 10.1016/s0092-8674(02)01047-4 PMC3499980

[B25] Piccioli Z. D. Littleton J. T. (2014). Retrograde BMP signaling modulates rapid activity-dependent synaptic growth via presynaptic LIM kinase regulation of cofilin. *J. Neurosci.* 34 4371–4381. 10.1523/JNEUROSCI.4943-13.2014 24647957 PMC3960475

[B26] Prokop A. Landgraf M. Rushton E. Broadie K. Bate M. (1996). Presynaptic development at the Drosophila neuromuscular junction: assembly and localization of presynaptic active zones. *Neuron* 17 617–626. 8893020 10.1016/s0896-6273(00)80195-6

[B27] Rui M. Qian J. Liu L. Cai Y. Lv H. Han J. (2017). The neuronal protein Neurexin directly interacts with the Scribble-Pix complex to stimulate F-actin assembly for synaptic vesicle clustering. *J. Biol. Chem.* 292 14334–14348. 10.1074/jbc.M117.794040 28710284 PMC5582829

[B28] Sen A. Yokokura T. Kankel M. W. Dimlich D. N. Manent J. Sanyal S. (2011). Modeling spinal muscular atrophy in Drosophila links Smn to FGF signaling. *J. Cell Biol.* 192 481–495. 10.1083/jcb.201004016 21300852 PMC3101100

[B29] Shi W. Chen Y. Gan G. Wang D. Ren J. Wang Q. (2013). Brain tumor regulates neuromuscular synapse growth and endocytosis in Drosophila by suppressing mad expression. *J. Neurosci.* 33 12352–12363. 10.1523/JNEUROSCI.0386-13.2013 23884941 PMC6618673

[B30] Sun M. Liu L. Zeng X. Xu M. Liu L. Fang M. (2009). Genetic interaction between Neurexin and CAKI/CMG is important for synaptic function in Drosophila neuromuscular junction. *Neurosci. Res.* 64 362–371. 10.1016/j.neures.2009.04.009 19379781

[B31] Sun M. Xing G. Yuan L. Gan G. Knight D. With S. I. (2011). Neuroligin 2 is required for synapse development and function at the Drosophila neuromuscular junction. *J. Neurosci.* 31 687–699. 10.1523/JNEUROSCI.3854-10.2011 21228178 PMC6623462

[B32] Sutcliffe B. Forero M. G. Zhu B. Robinson I. M. Hidalgo A. (2013). Neuron-type specific functions of DNT1, DNT2 and Spz at the Drosophila neuromuscular junction. *PLoS One* 8:e75902. 10.1371/journal.pone.0075902 24124519 PMC3790821

[B33] Tong H. Li Q. Zhang Z. C. Li Y. Han J. (2016). Neurexin regulates nighttime sleep by modulating synaptic transmission. *Sci. Rep.* 6:38246. 10.1038/srep38246 27905548 PMC5131284

[B34] Torroja L. Packard M. Gorczyca M. White K. Budnik V. (1999). The Drosophila beta-amyloid precursor protein homolog promotes synapse differentiation at the neuromuscular junction. *J. Neurosci.* 19 7793–7803. 10479682 10.1523/JNEUROSCI.19-18-07793.1999 PMC6782486

[B35] Tsuda H. Han S. M. Yang Y. Tong C. Lin Y. Q. Mohan K. (2008). The amyotrophic lateral sclerosis 8 protein VAPB is cleaved, secreted, and acts as a ligand for Eph receptors. *Cell* 133 963–977. 10.1016/j.cell.2008.04.039 18555774 PMC2494862

[B36] Tsuda H. Jafar-Nejad H. Patel A. J. Sun Y. Chen H. K. Rose M. F. (2005). The AXH domain of Ataxin-1 mediates neurodegeneration through its interaction with Gfi-1/Senseless proteins. *Cell* 122 633–644. 16122429 10.1016/j.cell.2005.06.012

[B37] Wu C. H. Giampetruzzi A. Tran H. Fallini C. Gao F. B. Landers J. E. (2017). A Drosophila model of ALS reveals a partial loss of function of causative human PFN1 mutants. *Hum. Mol. Genet.* 26 2146–2155. 10.1093/hmg/ddx112 28379367 PMC6251673

[B38] Xing G. Gan G. Chen D. Sun M. Yi J. Lv H. (2014). Drosophila neuroligin3 regulates neuromuscular junction development and synaptic differentiation. *J. Biol. Chem.* 289 31867–31877. 10.1074/jbc.M114.574897 25228693 PMC4231665

[B39] Xing G. Li M. Sun Y. Rui M. Zhuang Y. Lv H. (2018). Neurexin-Neuroligin 1 regulates synaptic morphology and functions via the WAVE regulatory complex in Drosophila neuromuscular junction. *eLife* 7:e30457. 10.7554/eLife.30457 29537369 PMC5873926

[B40] Zempel H. Mandelkow E. M. (2015). Tau missorting and spastin-induced microtubule disruption in neurodegeneration: Alzheimer disease and hereditary spastic paraplegia. *Mol. Neurodegener.* 10:68. 10.1186/s13024-015-0064-1 26691836 PMC4687341

[B41] Zhang P. Lu H. Peixoto R. T. Pines M. K. Ge Y. Oku S. (2018). Heparan sulfate organizes neuronal synapses through neurexin partnerships. *Cell* 174:1450-1464.e23. 10.1016/j.cell.2018.07.002 30100184 PMC6173057

[B42] Zhang X. Rui M. Gan G. Huang C. Yi J. Lv H. (2017). Neuroligin 4 regulates synaptic growth via the bone morphogenetic protein (BMP) signaling pathway at the Drosophila neuromuscular junction. *J. Biol. Chem.* 292 17991–18005. 10.1074/jbc.M117.810242 28912273 PMC5672027

[B43] Zito K. Parnas D. Fetter R. D. Isacoff E. Y. Goodman C. S. (1999). Watching a synapse grow: noninvasive confocal imaging of synaptic growth in Drosophila. *Neuron* 22 719–729. 10230792 10.1016/s0896-6273(00)80731-x

